# Impact of endometrial scratching on reproductive outcome in patients: A systematic review and meta-analysis

**DOI:** 10.1097/MD.0000000000030150

**Published:** 2022-08-19

**Authors:** YiFan Kang, ZhiHong Wang, Yuan Yang, HuiZhi Liang, Xia Duan, QingZhuo Gao, ZhaoFang Yin

**Affiliations:** Department of Reproductive Center, The First Hospital of Shanxi Medical University, Taiyuan, China.

**Keywords:** assisted reproductive technology, endometrial scratching, meta-analysis, outcome

## Abstract

**Background:**

Endometrial scratching (ES) has demonstrated initial success in women with recurrent implantation failure, but the effect in women with 1 previous assisted reproductive technology (ART) failure is unknown. This meta-analysis aimed to evaluate the impact of ES as a treatment in clinical outcomes for women with at least 1 failed in vitro fertilization (IVF)/intracytoplasmic sperm injection (ICSI)/Intrauterine Insemination (IUI).

**Methods:**

PubMed, Medline, Embase, Cochrane Library, Web of Science, CNKI, and EMCC databases were searched for randomized controlled trial studies utilizing endometrial scratching for infertility women with at least 1 failed assisted reproductive technology (ART) to collect pregnancy outcomes, including clinical pregnancy rate (CPR), embryo implantation rate (IR), miscarriage rate (MR), live birth rate (LBR), and multiple pregnancy rate (MPR).

**Results:**

Sixteen randomized controlled trial (RCT) studies were included in this meta-analysis, including 1770 women in the intervention group and 1934 women in the control group. Overall, the CPR, IR and LBR were significantly higher in the intervention group than in the control group (for CPR, n = 1430, 16 studies, *P* = .0002, risk ratio (RR) = 1.59, 95% confidence interval [CI] [1.24, 2.03]; for IR, n = 859, 10 studies, *P* = .0003, RR = 1.67, 95% CI [1.26, 2.21]; for LBR, n = 156, 6 studies, *P* = .0005, RR = 1.59, 95% CI [1.22, 2.06]). Nonetheless, there was no significant difference in MR (n = 344, 11 studies, *P* = .62, risk ratio (RR) = 0.92, 95% confidence interval [CI] [0.66, 1.29]) and MPR (n = 98, 3 studies, *P* = .39, risk ratio (RR) = 0.81, 95% confidence interval [CI] [0.51, 1.30]) between the intervention group and the control group.

**Conclusion:**

Endometrial scratching is considered to enhance the reproductive outcomes of embryo implantation. Additional randomized controlled studies are recommended to identify the appropriate time of invasion and the applicable population to confirm whether it can become a routine operation.

## 1. Introduction

Endometrial scratching (ES) is the intentional injury to the endometrium using instruments, most frequently a Pipelle catheter inserted through the uterus.^[1]^ This is a simple method that causes mild damage and has been proposed to improve pregnancy outcomes in assisted reproductive technology (ART). An animal study verified for the first time that endometrial scratching can enhance the pregnancy rate. Loeb reported in 1907 that endometrial injury provoked endometrial cell manipulation in guinea pigs,^[2]^ enhancing the uterine cavity environment and encouraging embryo implantation.

As the demand for assisted reproductive technology continues to increase, so does the pressure to improve success rates of in vitro fertilization (IVF)/intracytoplasmic sperm injection (ICSI)/Intrauterine Insemination (IUI). It is essential to determine strategies to enhance embryo implantation for all women undergoing ART. It is reported that the use of biopsy catheters for endometrial injury during the luteal phase of the menstrual cycle has been reported to improve implantation and pregnancy rates in subsequent treatment cycles.^[3]^ Endometrial scratching to alter the implantation window period for personalized embryo transfer has had a beneficial effect on treatment outcome,^[4]^ synchronizing the developing embryo with the receptive endometrium.^[5]^ Endometrial tolerance has been an important rate-limiting step in the success of in vitro fertilization, and its reduction is thought to be partly responsible for couples diagnosed with unexplained infertility,^[6]^ which is essential for achieving a viable pregnancy.

In 2003, Barash et al^[7]^ reported in a prospective randomized controlled trial that repeated local injury to the endometrium in the preIVF cycle doubled the incidence of successful pregnancy in 134 patients with a history of at least 1 cycle of IVF failure. A review provided endometrial curettage as the adjunctive technique with evidence of a high live birth rate.^[8]^ Furthermore, scratching the endometrium improves endometrial tolerance and increases embryo implantation and pregnancy rates.^[9]^

The potential benefit of endometrial scratching for embryo implantation remains a controversial topic. Endometrial scratching may enhance live birth rates in women with 2 or more failed in vitro fertilization.^[10]^ Women with repeated implant failures (RIF) have a strong desire to have a newborn child, so it is acceptable to perform traumatic operations and suffer pain.^[11]^ A recent systematic review reported that although endometrial scratching is associated with improved reproductive outcomes, more evidence is needed to demonstrate benefits for women undergoing their first or second in vitro fertilization cycle.^[12]^ So, researchers keep trying again and again.

With the generation of the latest randomized controlled trials now available, it is vital to update and summarize previous data. The main objective of this systematic review and meta-analysis is to confirm whether endometrial scratching is more effective in pregnancy outcomes in women with 1 or more previous failures of ART.

## 2. Materials and Methods

A research protocol was registered through PROSPERO: International Prospective Register of Systematic Reviews (protocol CRD42022296435) and completed conforming to the Preferred Reporting Items for Reviews and Meta-Analyses (PRISMA) guidelines for systematic review.

### 2.1. Literature search

Studies were identified through a systematic literature search on online databases: PubMed, Medline, Embase, Cochrane Library, Web of Science, CNKI, and EMCC. We performed an electronic database search for full-text articles and published abstracts from the inception of each database to December 2021. We did not limit the search by language, geographic origin, date of publication, or study type. Studies were limited to humans and animal studies were excluded. For database searches, the following main keywords were the following text words: “endometrial injury” or “local injury to endometrium” or “local endometrial injury” or “endometrial scratch” or “endometrial biopsy” or “endometrial damage” or “endometrial mechanical stimulation” [Mesh] AND “infertility” or “fertility” or “outcome” or “pregnancy” or “abortion” or “live birth” or “IVF” or “ICSI” or “IUI” or “FET” or “ART” or “artificial conception” or “embryo transfer” or “embryo implantation” or “endometrial receptivity”.

### 2.2. Inclusion and exclusion criteria

The criteria for inclusion were established before the literature search. All available randomized controlled trials that compared reproductive outcomes to the impact of local endometrial injury in patients were included. There were no restrictions on the stage of the embryo biopsy.

Inclusion criteria included women aged 18–43; body mass index (BMI) 18.5–32 kg/m^2^; normal hormone levels [follicle stimulating hormone (FSH), luteinizing hormone (LH), thyroid-stimulating hormone (TSH), testosterone (T), and prolactin (PRL)]; a normal uterine cavity and no adnexal masses; having experienced at least 1 failed in vitro fertilization cycle; more than 2 eligible embryos, at least 1 good quality embryo; undergone fresh or frozen embryo transfer, primary or secondary infertility due to unexplained or mild male factor.

Exclusion criteria included endometrial polyps; submucosal smooth muscle tumors; genital tract abnormalities (septum uterine, unicornuate uterus); intrauterine adhesions (Asherman syndrome); severe adenomyosis; moderate to severe pelvic endometriosis; women with low ovarian reserve: polycystic ovary syndrome (PCOS); genital tuberculosis unilateral or bilateral hydrocele; significant cardiovascular, pulmonary, renal, neurological or hepatic problems; sperm, gamete or embryo donor; chromosome abnormalities.

Moreover, the included studies had at least 1 of the following quantitative outcomes: clinical pregnancy rate (CPR), implantation rate (IR), miscarriage rate (MR), multiple pregnancy rates (MPR) and live birth rate (LBR).

### 2.3. Study selection and data extractions

From the literature search, 818 abstracts of studies were retrieved and independently screened for inclusion. The information extracted included study general information (title, author, year, and journal), study characteristics (type of study design, outcome), and interventions. We excluded 802 abstracts for any 1 of the following reasons: nonrelevant material, abstracts only, animal studies, trial registry abstracts without published data, duplicate abstracts, or review articles. 16 full-text articles were reviewed for inclusion. Furthermore, all authors reviewed studies if there was a disagreement about inclusion. All studies met the inclusion criteria and were subsequently reviewed and analyzed.

**Figure 1.**
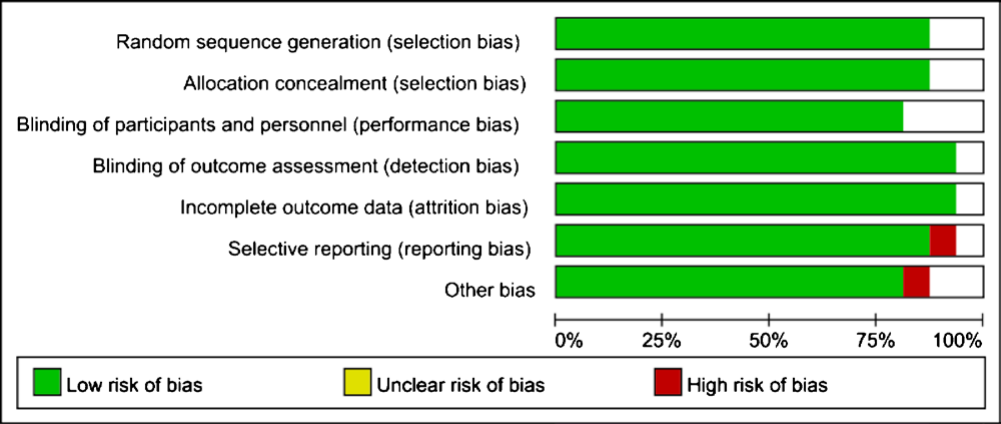
Risk of bias graph.

The authors independently implemented the Cochrane Risk of Bias Assessment Tool to assess for the following biases: selection (random sequence generation, allocation concealment), performance (blinding of participants and personnel), detection (blinding of outcome assessment), attrition (incomplete outcome data), reporting (selective reporting), and other (not otherwise specified). All judgments were reported as “low risk,” “high risk” or “unclear risk” of bias. As all of the incorporated studies were randomized controlled trials, the risk of bias was mostly low in the RCTs (Fig. 1). Consequently, the quality of evidence for this study is high.

### 2.4. Quality assessment and statistical analysis

All meta-analyses were performed using Review Manager 5.3 (Cochrane Collaboration, Oxford, UK). Continuous and dichotomous variables were analyzed using weighted mean differences (WMDs) and risk ration (RRs) with 95% confidence intervals (CIs) respectively. The statistical heterogeneity was quantified using the I^2^. The random-effects model was used if there was heterogeneity between studies(I^2^ > 50%); otherwise, the fixed-effects model was used(I^2^<50%).

Sensitivity was performed by excluding and calculating each study from the meta-analysis 1 at a time and counting the pooled effect size to determine the influence of each study. Publication bias was screened using funnel plots. A *P* < .05 was considered statistically significant.

### 2.5. Ethics and dissemination

Ethics approval was not required for this systematic review because of the data used does not include personal data. Therefore, there were no concerns about privacy.

## 3. Results

### 3.1. Selection of studies for inclusion in systematic review and meta-analysis

**Figure 2.**
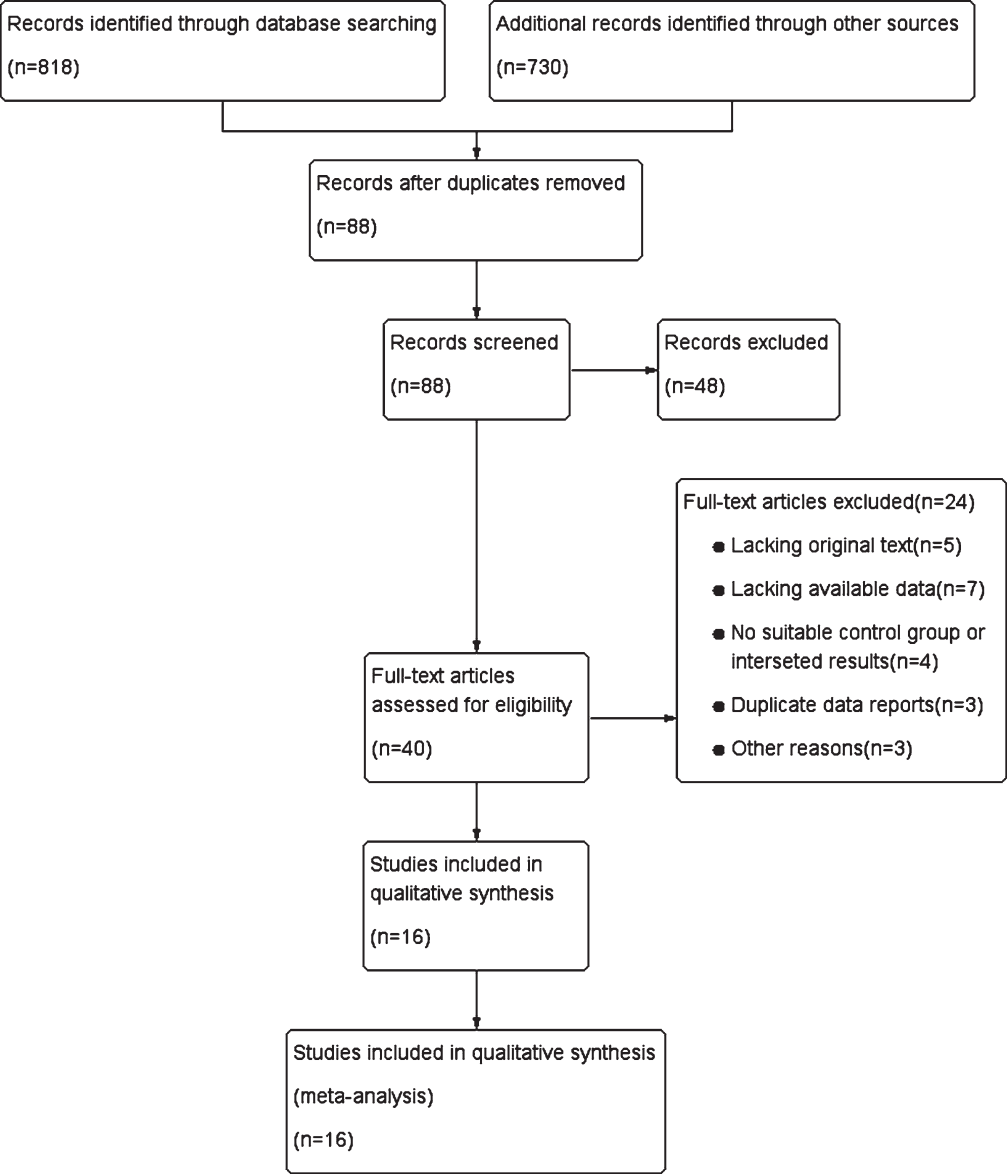
Flowchart of literature search and study inclusion.

The detailed study selection process is documented in the PRISMA (Preferred Reporting Items for Systematic Reviews and Meta-Analyses) flowchart. The search strategy is illustrated in Figure 2. The initial systematic literature search yielded 818 publications. After assessing the full text of 40 articles, 24 studies were excluded.

16 papers were ineligible for the following reasons: 12 papers did not provide complete data for this meta-analysis, 4 papers without a control group and available results, 3 papers with replicated data reports, and 3 papers for other reasons. Ultimately, 16 studies met the screening criteria and were included in this meta-analysis (Table 1). Overall, a total of 1430 patients were randomly assigned to either the ES or nonES group for these studies.

**Table 1:** Characteristics of the included studies.

Study	Design	Intervention	Participants	Timing of intervention	Outcomes
Ashrafi et al 2017	RCT	Intervention:pipeline biopsy;control:no biopsy	Previous failed ≥ 1 IUI, normal uterine anatomy and hysterosalpingography	Preceding cycle days 8 or 9	CPR, miscarriage rate
Barash et al 2003	RCT	Intervention: pipeline biopsy 4 times; control: no biopsy	Previous failed ≥ 1 IVF-embryo transfer, good responders to hormonal stimulation, age 23–45	Preceding cycle days 8, 12, 21 and 26	CPR, IR, LBR, miscarriage rate
Baum et al 2012	RCT	Intervention: pipeline biopsy twice; control: no biopsy but adding cervical pipeline	Previous failed ≥ 3 fresh IVF cycles, good responders, age 18–41	Preceding cycle days 9–12 and 21–24	IR, CPR, LBR
Berntsen et al 2020	RCT	Intervention: pipeline biopsy twice; control: no biopsy	Previous failed ≥ 1 IVF/ICSI cycle, age 18–40		CPR, LBR
Chen et al 2013	RCT	Intervention: pipelle biopsy; control: no biopsy	Previous failed ≥ 3 fresh IVF cycles, good responders, age ≤ 40	Preceding cycle days 3–7	IR, CPR
Frantz et al 2018	RCT	Intervention: pipeline biopsy; control: no biopsy	Previous failed ≥ 1 IVF/ICSI cycle, age 18–38	Preceding cycle days 20 or 24	CPR, IR, multiple pregnancy rate
Gibreel et al 2015	RCT	Intervention: pipeline biopsy twice; control: no biopsy	Previous failed ≥ 1 IVF cycle, age < 40, good responders, normal uterine cavity	On day 21 of the preceding IVF cycle and then after2–3 days	CPR, LBR, miscarriage rate, multiple pregnancy rate
Karimzadeh et al 2009	RCT	Intervention: pipeline biopsy; control: no biopsy	2–6 unsuccessful IVF-embryo transfer, transfer of ≥ 10 high-grade embryos, age 20–40, good responders	Preceding cycle luteal phase days 21–26	CPR, IR
Lei et al 2018	RCT	Intervention: pipeline biopsy; control: no biopsy	Previous failed ≥ 1 IVF/ICSI cycle, age 20–40	Embryo-transfer cycle days 2–3	CPR, IR, LBR, miscarriage rate, multiple pregnancy rate
Levin et al 2017	NR	Intervention: pipeline biopsy first to twice; control: no biopsy	Previous failed ≥ 1 IVF/ICSI cycle	In the proliferative phase and the secretory phase of the spontaneous the menstrual cycle before the index IVF-ET treatment	CPR, miscarriage rate
Narvekar et al 2010	RCT	Intervention: pipeline biopsy twice; control: no biopsy	Previous failed ≥ 1 cycle with good-quality embryos, good responders, age ≤ 37, HS normal cavity	Preceding cycle days 7–10 and 24–25	LBR, IR, CPR, miscarriage rate
Olesen et al 2019	RCT	Intervention: pipeline biopsy; control: no biopsy	Previous failed ≥ 1 IVF/ICSI cycle, age 18–40	Preceding cycle days 18–22	CPR,LBR,miscarriage rate, multiple pregnancy rate
Several et al 2016	RCT	Intervention: pipeline biopsy; control: no biopsy	Previous failed ≥ 2 IVF/ICSI or FET cycles, age 20–40	Preceding cycle days 7–14	CPR, IR, miscarriage rate, multiple pregnancy rate
Van et al 2021	RCT	Intervention: pipeline biopsy; control: no biopsy	Previously failed one IVF/ICSI cycle,age 18–44	Preceding cycle days 5–10	CPR, miscarriage rate
Wen et al 2021	RCT	Intervention: pipeline biopsy; control: no biopsy	Previous failed ≥ 2 IVF/ICSI cycles, age ≥ 18	Preceding cycle days 7–14	CPR, IR
Zhao et al 2017	RCT	Intervention: pipeline biopsy; control: no biopsy	Previous failed 1 IVF/ICSI cycle, age ≤ 40	Preceding cycle days 3–7	CPR, IR, miscarriage rate, multiple pregnancy rate

### 3.2. Pregnancy outcomes

**Figure 3.**
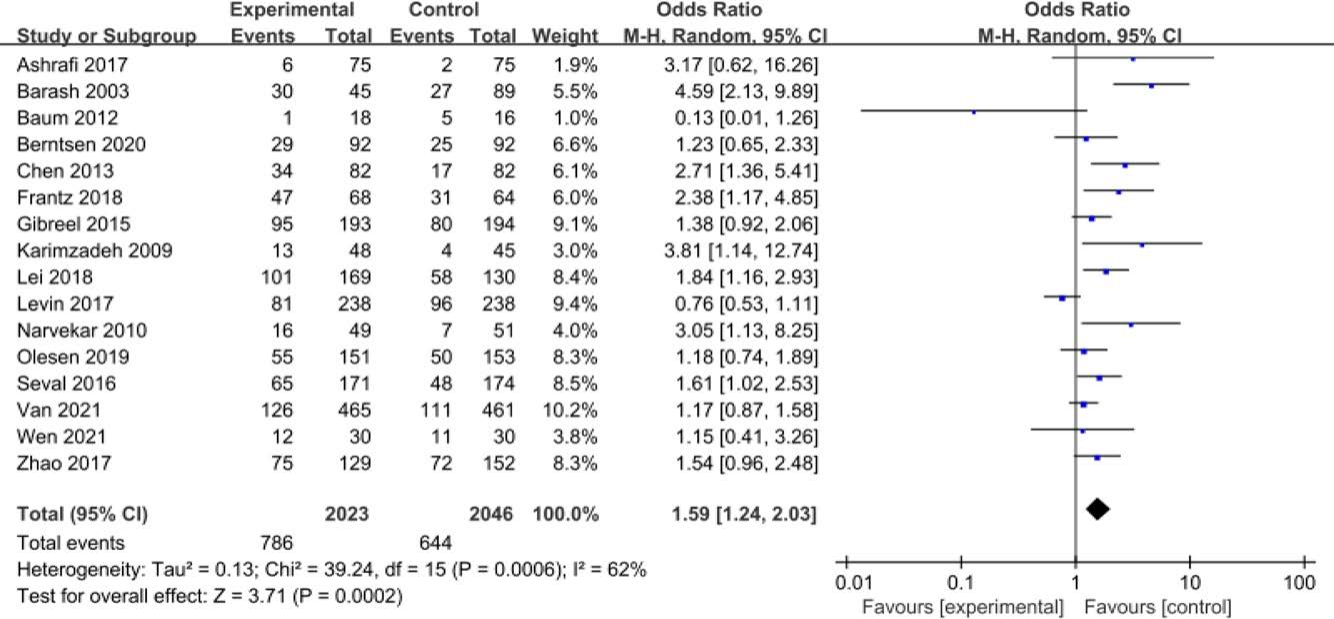
Forest plot graphs for clinical pregnancy rate in the endometrial biopsy and control groups. Random-effects model was used in this meta-analysis, the risk ratio was used to measure the effect size. CI = confidence interval, CPR = clinical pregnancy rate, IR = implantation rate, LBR = live birth rate, MPR = multiple pregnancy rate, MR = miscarriage rate.

**Figure 4.**
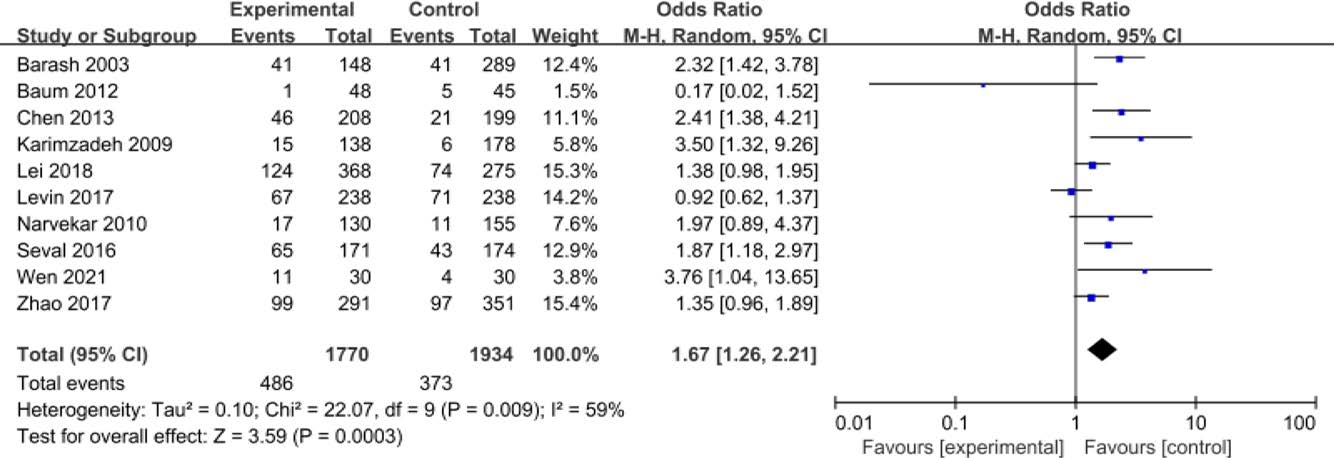
Forest plot graphs for implantation rate in the endometrial biopsy and control groups. Random-effects model was used in this meta-analysis; the risk ratio was used to measure the effect size. CI = confidence interval, CPR = clinical pregnancy rate, IR = implantation rate, LBR = live birth rate, MPR = multiple pregnancy rate, MR = miscarriage rate.

**Figure 5.**
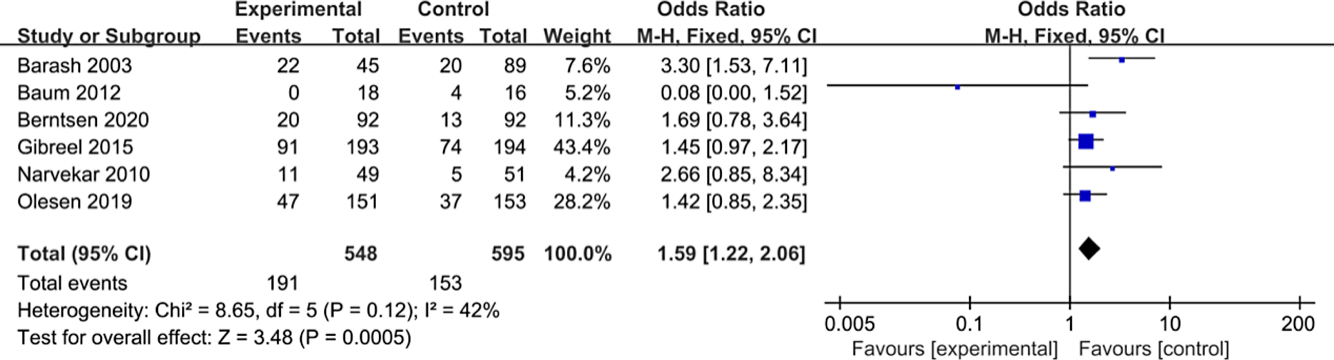
Forest plot graphs for live birth rate in the endometrial biopsy and control groups. Random-effects model was used in this meta-analysis, the risk ratio was used to measure the effect size. CI = confidence interval, CPR = clinical pregnancy rate, IR = implantation rate, LBR = live birth rate, MPR = multiple pregnancy rate, MR = miscarriage rate.

**Figure 6.**
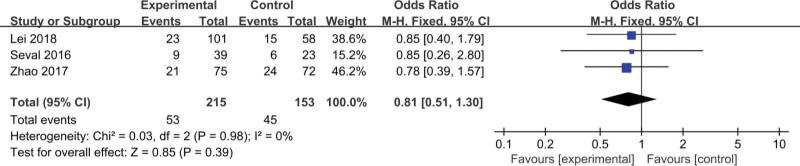
Forest plot graphs for miscarriage rate in the endometrial biopsy and control groups. Random-effects model was used in this meta-analysis, the risk ratio was used to measure the effect size. CI = confidence interval, CPR = clinical pregnancy rate, IR = implantation rate, LBR = live birth rate, MPR = multiple pregnancy rate, MR = miscarriage rate.

This article compares assisted reproduction outcomes in the ES and nonES groups (Figures 3–6). Pooled analysis revealed that 16 studies reported a total of 1430 clinical pregnancies with an increased pregnancy rate in the ES group (RR 1.59, 95% CI 1.24–2.03, *P* = .0002). 10 studies reported embryo implantation rates with a total of 859 patients with a significant difference seen (RR 1.67, 95% CI 1.26–2.21, *P* = .0003). Six studies reported (including 156 patients) a higher live birth rate in the ES group (RR 1.59, 95% CI 1.22–2.06, *P* = .0005) than in the nonES group. 11 studies (including 344 patients) compared the miscarriage rate in the ES and nonES groups and the difference was not statistically significant (RR 0.92, 95% CI 0.66–1.29, *P* = .62). Three studies (including 98 patients) reported no significant difference in multiple pregnancy rates (RR 0.81, 95% CI 0.51–1.30, *P* = .39) between the 2 groups.

### 3.3. Sensitivity analyses

Studies assessing pregnancy and embryo implantation rates by Q test and I^2^ index demonstrated high heterogeneity (*P* = .0006, I^2^ 62%), (*P* = .009, I^2^ 59%). Assessment of miscarriage rate (*P* = .12, I^2^ 42%), live birth rate (*P* = .82, I^2^ 0%) and multiple pregnancy rate (*P* = .98, I^2^ 0%) showed low heterogeneity. An example is indicated by sensitivity analysis showing the funnel plot of the miscarriage rates reported in this meta-analysis for the ES and nonES groups (Fig. 8).

**Figure 7.**
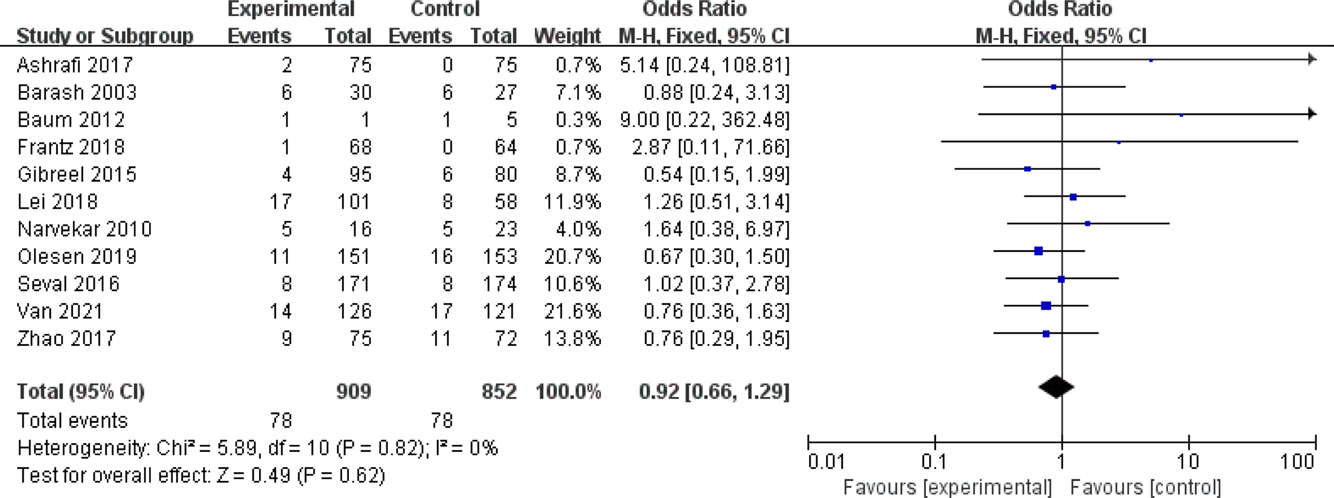
Forest plot graphs for multiple pregnancy rates in the endometrial biopsy and control groups. Random-effects model was used in this meta-analysis, the risk ratio was used to measure the effect size. CI = confidence interval, CPR = clinical pregnancy rate, IR = implantation rate, LBR = live birth rate, MPR = multiple pregnancy rate, MR = miscarriage rate.

**Figure 8.**
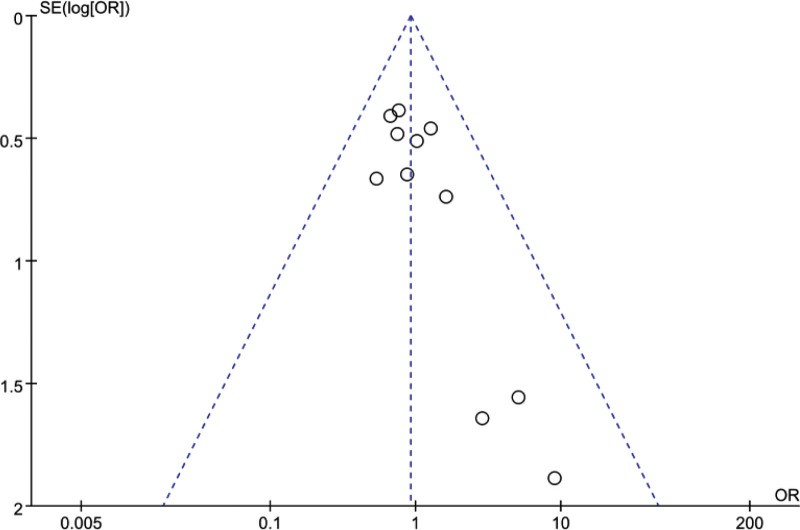
Funnel plot assessment of publication bias.

## 4. Discussion

The results of this systematic review and meta-analysis suggested that performing ES in infertile women who have failed at least 1 IVF/ICSI/IUI improves pregnancy rate, embryo implantation rate, and live birth rate without increasing miscarriage or multiple pregnancy rates, which successfully improves pregnancy outcomes. The survey sought to confirm the true effectiveness of ES in upgrading endometrial tolerance.

ART can overcome many of the causes of infertility, which is used to salvage the barriers to implantation failure and to improve the chances of successful implantation.^[13]^ The implantation process consists of 2 main components: a healthy embryo and an endometrium that is easy to implant.^[14]^ The mid-secretory phase of the menstrual cycle (day 19–23), the period when the endometrium is most receptive, is known as the implantation window period.^[15]^ Barash et al^[7]^ first proposed endometrial scratching or injury as a simple intervention to upgrade endometrial tolerance in patients undergoing ART. They found that intervention of the endometrium on days 8 and 12 of the menstrual cycle was associated with higher pregnancy rates after IVF, which positively impacted endometrial tolerance. Accordingly, for the last 2 decades, there has been expanding global interest in exploring whether mechanical stimulation of the endometrium can be utilized as a method to improve endometrial receptivity.

Endometrial scratch is an intentional injury to the endometrium in infertile women, usually using a tube inserted into the uterine cavity to remove a sample of the endometrium by rotation and aspiration. This is a simple and low-cost procedure that can cause minor discomfort and pain. It has been observed that local damage to the endometrium of rodents causes rapid growth of meconium cells.^[16]^ Another study has stimulated the growth of endometrial cells comparable to gestational meconium cells caused by oil injection in animals.^[17]^ The underlying mechanisms by which ES improves endometrial tolerance remain unclear, and there are currently 2 theories hypothesizing how endometrial injury operates.

One hand is that mechanical trauma can delay endometrial maturation by inducing the conversion of proliferative endometrium to metaphase,^[17,18]^ which overcomes the synchronization disorder caused by controlled ovarian hyperstimulation (COH) and thus restores embryo-endometrial synchronization.^[19,20]^ On the other hand, the inflammation of local damage aggregate signal local immune cells, upregulating cytokines necessary for embryo implantation, such as tumor necrosis factor-α (TNF-α), interleukin-15 (IL-15), growth regulatory oncogene-α (GRO-α), macrophage inflammatory protein-1B (MIP-1B).^[21]^ The inflammatory factors are vital for embryo implantation,^[22]^ which spark wound healing, improve endometrial maturation^[23]^ and promote embryo implantation.^[24]^ In addition, ES can alter the gene expression of certain distinct factors, such as laminin 4, integrin 6, matrix metalloproteinase 1, and glycosidic protein A,^[23]^ which benefited embryo implantation.^[24]^ In addition to the above theories, local damage to the endometrium during COH cycles causes significant differences in the expression of messenger RNA in the endometrium.^[25,26]^ MicroRNAs are involved in the implantation process in several ways^[27]^ but are not clearly explained.

Research to date indicates that despite the lack of strong evidence, scratching the endometrium still shows beneficial effects. In this respect, localized endometrial injury in patients during the COH cycle raised embryo implantation rate, clinical pregnancy rate, and live birth rate, with no significant effect on miscarriage or multiple pregnancy rate. It is consistent with the results of Zhou et al^[28]^ and Van et al^[29]^ During ovarian stimulation, high levels of estradiol can cause a premature rise in progesterone, leading to an early window period and preventing implantation, thus ES in the preIVF cycle may improve the intrauterine environment.^[30]^ Gibreel et al^[31]^ and Parsanezhad et al^[32]^ similarly concluded that endometrial damage is beneficial for pregnancy outcomes in infertile women undergoing IVF/ICSI. Moreover, endometrial damage in IUI cycles also improves pregnancy rates.^[33]^ Furthermore, while some studies have not revealed significant beneficial effects, no negative effects of ES have been observed.^[34]^ Hence, it can be momentarily considered a nondestructive operation.

Logistic regression analysis in 2017^[35]^ found that first ES was not significantly associated with pregnancy rate. Mechanical endometrial stimulation did not increase the rate of implantation or pregnancy rate. In addition, no factors were discovered that could predict which patients would benefit from ES. Izquierdo et al^[36]^ and Frantz et al^[37]^ similarly concluded that ES does not improve the clinical outcome of first in vitro fertilization. During the same period, no significant difference in reproductive outcomes was also observed between the ES and control groups in women who had at least 1 failed IVF/ICSI cycle in another RCT.^[38]^ In a historically controlled cohort study, ES was found to be useful in women with repeated failed implantation.^[13]^ Gibreel^[11]^ demonstrated that endometrial scratching may improve the live birth rate in women with 2 or more failed in vitro fertilization, which is an independent predictor. The results remain contentious. Hence, scientists have attempted to employ ES in women with 1 or more previous failures to observe changes in clinical outcomes.^[21]^

Although this intervention may benefit patients with 2 or more IVF failures, its effectiveness in infertile women without previous failures remains unclear. Local endometrial injury is not recommended in women undergoing the first ART until guidelines or joint expert guidance is presented,^[39]^ but can be used in women with RIF. ES may be 1 of the secret weapons of IVF fertility success for RIF patients.^[40]^ The 2014 Cochrane database affirmed it as moderate-quality evidence.^[41]^ A recent systematic review reported that although endometrial scratching is associated with improved reproductive outcomes, more evidence is required to demonstrate benefits for women undergoing their first or more in vitro fertilization cycle.^[10]^ A couple who underwent 1 failed IVF/ICSI therapy scratched the endometrium and underwent a second IVF/ICSI, improving their live birth rate by 4.6%.^[42]^ This article proved that ES during ART is considered to enhance reproductive outcomes which are medium-quality evidence to support the above conclusion.

The optimal timing of ES is discussed in depth. Endometrial scratching can be executed between the previous cycle and day 7 of the embryo transfer cycle.^[1]^ Several scholars verified that ES performed 7–14 days before the ART cycle is favorable for pregnancy outcomes.^[24]^ Several scholars confirmed that ES performed 7–14 days before the ART cycle is beneficial for pregnancy outcomes.^[43]^ It may inhibit proliferation and thus optimize synchronization between the endometrium and the transferred embryo.^[17]^ It is also discovered that no deleterious effects were observed when ES was executed in the same stimulation cycle.^[34]^ The prompt ES time is uncertain. There is also some evidence that adverse effects can be observed with biopsies executed directly before embryo transfer.^[44]^ The operation is performed shortly before the onset of embryo transfer, resulting in the formation of pro-inflammatory cytokines, macrophages and dendritic cells not fast enough and not in sufficient numbers. Consequently, the endometrial tolerance is compromised rather than enhanced.^[45]^ The scratching times included in this study are not uniform, most of which are carried out in the previous cycle and show good pregnancy outcomes. We recommend that the procedure be performed as soon as possible to improve the results of ART.

Further studies have found that the pro-inflammatory environment is caused by the levels of endometrial cytokines (MIP-1B, IL-15, TNF, VEGF) or the accumulation of endometrial immune cells (DCs, macrophages, uNKs). After scratching these factors may facilitate the process of implantation by facilitating communication between the embryo and the endometrium and by reducing the negative effects of ovarian stimulation on the endometrium.^[29]^ The selection of appropriate endometrial cytokines or immune cells can be a biomarker of local immune activation or inflammation. Dr Cavalcante et al^[29]^ concluded that endometrial scratching is most likely to benefit only those patients with specific underlying pathologies characterized as endometrial immune biomarkers. Therefore, they suggested that future studies should contain endometrial immune biomarkers as a criterion. Scratching during ART in patients with recurrent implantation failure appears to verify this therapeutic effect, as they consistently show varying degrees of immune compromise. The comparison revealed statistically significant differences in messenger RNA expression profiles. Further studies of these genes will also help predict implantation capacity.

Research indicates that scratching seems to be a measure of success in upgrading the pregnancy rate of women with failed implantation.^[46–48]^ We aim to highlight some of the research directions proposed for achieving of a one-time pregnancy in ART to investigate the clinical utility of targeted endometrial scratching. There is insufficient evidence to support the use of endometrial scratching in the treatment of ART and no consensus has been established regarding the efficacy, safety, and optimal timing of this intervention.

## 5. Limitations

One limitation of this paper is that there is no unified standard for the method, intensity, operation time and frequency of endometrial mechanical stimulation. Another limitation is that there is still no uniform standard for infertile women undergoing ES. After careful consideration, the results of this study do not apply to patients with low or high ovarian responses. In addition, the research did not classify endometrial thickness, and poor, thick, or thin endometrial morphology usually affects its receptivity to a certain extent, which is not conducive to embryo implantation. Consequently, it is difficult to determine to what extent stimulation of endometrial receptivity can trigger the improvement of endometrial receptivity and promote the synchronization of endometrial development. Whether endometrial stimulation is suitable for all infertile patient needs to be carefully explored, and the next step should be to find out which population is more benefited from ES, such as the number of assisted reproductive failures, endometrial morphology, and thickness, etc

## 6. Conclusion

The findings of this systematic review and meta-analysis demonstrated that endometrial scratching during ART is seen to improve reproductive outcomes by inducing beneficial changes in the endometrium to improve embryo implantation success. The people add were women who had failed ART in the past, but the reason for the failure is still unclear. It was highly considered that it was due to endometrial problems, rather than fallopian tube factors, reproductive tract abnormalities, or male factors. Accordingly, this survey can draw convincing conclusions, provide effective evidence for evidence-based medicine, and improve patient satisfaction, which is worthy of clinical promotion and treatment guidance. Additional randomized controlled studies are proposed to identify the appropriate time of the invasion, the applicable population, and whether it can become a routine means of operation.

## Acknowledgments

We are thankful to all the authors and all the study participants in this study.

## Author contributions

The authors’ contributions are as follows: conceived and designed the experiments, carried out the experiments, evaluated the data, and wrote and interpreted the paper.
